# Association of the Joint Effect of Menopause and Hormone Replacement Therapy and Cancer in African American Women: The Jackson Heart Study

**DOI:** 10.3390/ijerph8062491

**Published:** 2011-06-23

**Authors:** Brenda W. Campbell Jenkins, Clifton Addison, Gregory Wilson, Jiankang Liu, Melody Fortune, Kiana Robinson, Monique White, Daniel Sarpong

**Affiliations:** 1 Jackson Heart Study Coordinating Center, Jackson State University, 350 West Woodrow Wilson Drive, Suite 701, Jackson, MS 39213, USA; E-Mails: Clifton.addison@jsums.edu (C.A.); gregory.wilson@jsums.edu (G.W.); kiana.r.robinson@jsums.edu (K.R.); dannys107@yahoo.com (D.S.); 2 Jackson Heart Study, Department of Medicine, University of Mississippi Medical Center, 350 West Woodrow Wilson Drive, Suite 701, Jackson, MS 39213, USA; E-Mail: jliu@umc.edu; 3 Mississippi Department of Health, 570 East Woodrow Wilson, Jackson, MS 39213, USA; E-Mail: melody.fortune@msdh.state.ms.us; 4 Project Health, Jackson State University, 350 West Woodrow Wilson Drive, Suite 701, Jackson, MS 39213, USA; E-Mail: mswhite427@yahoo.com

**Keywords:** cancer, breast cancer, hormone replacement therapy, pre and post menopause, African Americans, Jackson Heart Study, joint effect, association

## Abstract

Cancer is the second leading cause of death in the US and in Mississippi. Breast cancer (BC) is the most common cancer among women, and the underlying pathophysiology remains unknown, especially among African American (AA) women. The study purpose was to examine the joint effect of menopause status (MS) and hormone replacement therapy (HRT) on the association with cancers, particularly BC using data from the Jackson Heart Study. The analytic sample consisted of 3202 women between 35 and 84 years of which 73.7% and 22.6% were postmenopausal and on HRT, respectively. There were a total of 190 prevalent cancer cases (5.9%) in the sample with 22.6% breast cancer cases. Menopause (p < 0.0001), but not HRT (p = 0.6402), was independently associated with cancer. Similar results were obtained for BC. BC, cancer, hypertension, type 2 diabetes, prevalent cardiovascular disease, physical activity and certain dietary practices were all significantly associated with the joint effect of menopause and HRT in the unadjusted analyses. The family history of cancer was the only covariate that was significantly associated with cancer in the age-adjusted models. In examining the association of cancer and the joint effect of menopause and HRT, AA women who were menopausal and were not on HRT had a 1.97 (95% CI: 1.15, 3.38) times odds of having cancer compared to pre-menopausal women after adjusting for age; which was attenuated after further adjusting for family history of cancer. Given that the cancer and BC cases were small and key significant associations were attenuated after adjusting for the above mentioned covariates, these findings warrant further investigation in studies with larger sample sizes of cancer (and BC) cases.

## 1. Introduction

Medical research has reported that cancer follows cardiovascular disease as the second leading cause of death, claiming the lives of over half a million adults annually [1]. Breast cancer is considered to be the most common form of cancer and the second leading cause of death among women in the United States. It is estimated that there could be more than 14,330 new cases of cancer in Mississippi and 1,529,560 cases in the U.S. In 2009, it was estimated that there would be 192,370 new diagnoses of invasive breast cancer and approximately 40,170 women are expected to die from breast cancer [2]. African American women are disproportionately represented in poor survival outcomes, and cancer mortality [3–6].

The Jackson Heart Study was initiated as a response to American health disparities in the US with a view to gaining a better understanding of key factors in the development of cardiovascular disease in African Americans [7]. Few studies have examined disease risks in a setting where data on risk factors for CVD have been collected along with participant information regarding the prevalence and incidence of different types of cancers. The Jackson Heart Study, therefore, has provided a unique opportunity for researchers to examine cancer status in relation to traditional and emerging CVD risk factors. In this paper, we examined the association of the joint effect of menopause and hormone replacement therapy and cancer in African American women in the Jackson Heart Study.

## 2. Study Design and Methods

The Jackson Heart Study (JHS) is a prospective cohort study of cardiovascular disease in non-institutionalized African American adults aged 21–95 residing in the Jackson, MS metropolitan area (MSA). The JHS is the largest single-site prospective epidemiologic investigation of cardiovascular disease in African Americans. The state of Mississippi has the highest percentage of African American residents (36.9%) of any state in the U.S. Participants were recruited from urban and rural areas of three counties in the Jackson MSA, which includes Hinds, Madison, and Rankin counties. Overall, 17% of JHS participants were recruited through random sampling of the Jackson MSA commercial database (Accudata), 22% as family members, 31% from the Jackson, MS site of the National Heart, Lung, and Blood Institute’s Atherosclerosis Risk in Communities study, and 30% as volunteers. The final JHS cohort included 5,301 participants, equivalent to 6.6% of all African Americans residing in the Jackson MSA. Data utilized in the analysis were obtained from JHS examination 1 (2000–2004) and the JHS examination 1 annual follow-up interview which was completed within three months of the anniversary date of the original visit. Study design has been detailed elsewhere [8–14].

The analytic sample size for this study is 3202 and it was derived using the exclusion criteria: a) exclusion of all male participants and b) exclusion of female participants who were 21–34 and 85–95 years old to minimize the potential of have a highly correlated data set, since these women were strictly recruited for the family study. The sample size for the analysis involving the joint effects of menopause and HRT was 3142 due to further exclusion of women who were pre-menopausal on HRT. The JHS variables utilized in this study were grouped into the following: outcome measures, reproductive measures, demographic and socioeconomic measures, clinical risk factors and behavioral risk factors, which included dietary practices.

### Outcome Measures

The primary and secondary outcome variables were prevalent cancer and prevalent breast cancer, respectively. Both prevalent cancer and breast cancer cases were defined by the participants responses to the following questions: (1) “Has your doctor or health care professional ever said you have cancer?” This question was taken from the Personal and Family History Questionnaire; (2) “Has a doctor ever said you had cancer?”; or (3)“Have you had another cancer?” Questions two and three were taken from the annual follow-up questionnaire. The date of diagnosis was prior to the participant’s JHS Exam 1 visit date.

### Reproductive Measures

Women who responded “yes”, at the baseline examination, to having had menstrual periods or bleeding during the past two years were classified as pre menopausal. Women who responded “no” to the same question were classified as post menopausal. Women were classified as currently taking HRT, if they responded “yes” to either of the following questions: are you currently taking: (a) first identified hormone, (b) second identified hormone or (c) third identified hormone in the reproductive history questionnaire?, and provided proof of hormone use based on medications that were brought to clinic visit. Medications were transcribed and coded as HRT by a pharmacist using the Medispan dictionary and classified according to the Therapeutic Classification System. History of contraceptive use was ascertained based on the history of taking birth control pills included in the JHS Reproductive History Questionnaire.

### Demographic and Socioeconomic Measures

Demographic information, including age and gender were obtained at the examination. Socioeconomic status was measured by annual family income. Income was self reported in the following categories: less than $5,000; $5,000–7,999; $8,000–11,999; $12,000–15,999; $16,000–19,999; $20,000–24,999; $25,000–34,999; $35,000–49,999; $50,000–74,999; $75,000–99,999; $100,000 or more. Income was classified as low, lower-middle, upper-middle, and high based upon family size, number of children <18 years of age and the U.S. Census designated poverty level for the year in which the income information was obtained. Low income was defined as below the poverty level for the corresponding family size/number of children combination. High income was defined as more than four times the poverty level threshold for each family size by child grouping. The two middle income categories were divided at 2.5 times the poverty level.

### Clinical Risk Factors

The clinical risk factors for cardiovascular disease examined in this paper included hypertension, diabetes, and obesity. Standardized questionnaires were used to obtain medical history information and medication usage within the past two weeks of exam 1 for diabetes and high blood pressure, and participants were asked to bring their medications to the examination. To determine hypertension status, two resting blood pressure readings were taken one minute apart at the exam using a Hawksley random-zero sphygmomanometer (Hawksley and Sons Ltd.) and averaged. Hypertension was defined according to JNC VII criteria as systolic blood pressure ≥140 mmHg or diastolic blood pressure ≥90 mmHg at exam, or use of blood pressure lowering medication (self-report and actual) within 2 weeks prior to the examination, or self-reported history of hypertension.

Fasting blood samples were also taken at the exam and blood glucose was measured at a central laboratory. Type II diabetes was defined according to American Diabetes Association 2004 criteria as fasting glucose ≥126 mg/dL, or confirmed medication inventory or self-reported use of anti-diabetic medications, or self-reported diabetes diagnosis. Body mass index (BMI) was derived to determine overweight/obesity status. BMI was calculated in kg/m^2^ using measurements of weight and height at the exam while participants wore light clothing and no shoes. Obesity was defined according to the World Health Organization standard of BMI ≥ 30.

### Behavioral Risk Factors

Current smoking status was self-reported by participants at the exam as having smoked 400 cigarettes or more in his/her lifetime and having smoked within 3 months of the JHS baseline interview. Heavy alcohol drinkers were defined as persons who consumed greater than 24 grams of alcohol per day. Physical activity was assessed using the Jackson Heart Study Physical Activity Cohort instrument derived from modification of the Baecke physical activity survey [15]. Total physical activity was computed as a summary score of the intensity, frequency, and duration of activities associated with: active living, including transportation and leisure time activities; home, family, yard, and gardening activities; occupational activities, and sport participation. The summary score was validated against results from 24-hour accelerometer and pedometer monitoring. Dietary measures used in this study were determined from participant responses to the JHS food frequency short-form questionnaire developed in conjunction with the Human Nutrition Research Center on Aging at Tufts University and the Delta Nutrition Intervention Research Initiative (sponsored by the US Department of Agriculture). The validation and calibration of the JHS food frequency short-form questionnaire are detailed by [16].

### Data Analytic Plan

Descriptive statistics were used to describe the characteristics of the study sample. Two-sample t-tests were performed to compare cancer cases and breast cancers cases with their respective controls for all the demographic, SES, lifestyle and clinical factors which were continuous in nature. For the categorical factors chi-square tests were performed to examine the association of the factors with prevalent cancer and prevalent breast cancer. These chi-square tests were performed for the purpose of identifying potential cofounders to be adjusted for in further analyses. Also, chi-square tests were used to examine the association of prevalent cancer and breast cancer with the joint effect of menopause status and hormone replacement therapy (HRT). The general linear modeling (GLM) approach was used to assess differences among the three groups defined by the joint effect of menopause and HRT. The test of association between prevalent cancer and breast cancer and the joint effect of menopause and HRT was conducted by means of multiple (multivariable-adjusted) logistic regression; which computed the odds ratios (OR) and 95% confidence intervals (CI). Age and covariates that were significantly associated with both outcome measures were included in each of the multiple logistic regression models. A significance level of 0.05 was used in all inferential analyses.

## 3. Results

**Figure 1.**
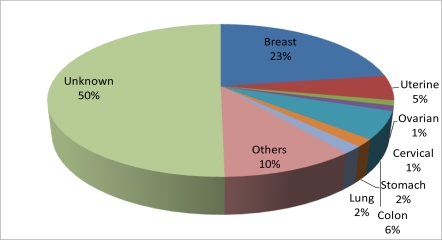
Distribution of types of cancer among women (35–84 years) in JHS (n = 190).

**Figure 2.**
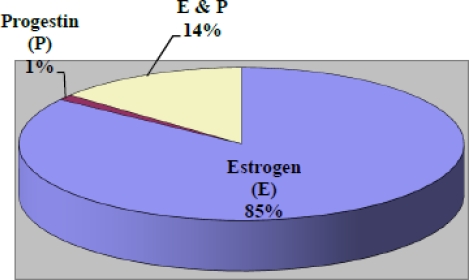
Distribution of types of hormone replacement therapy among women (35–84 years) in JHS (n = 1,216).

Table 1 below summarizes the demographic, medical and reproductive histories of the women in the Jackson Heart Study included in this study. The mean (standard deviation [SD]) age of the analytic sample was 56 (11) years with a mean body mass index of 32.8 (SD = 7.4) kg/m^2^. Generally, the study participants were well educated with approximately 61% reporting greater than a high school education; and 55.7% having a family income that placed them in the upper-middle to affluent income class. Their health profile concerning medical and reproductive history suggested that about 60% had a family history of cancer, 73.7% were post menopausal and 22.6% reported use of hormones. The mean (SD) number of pregnancies and live-born children were 3.5 (2.5) and 3.2 (2.2), respectively. There were a total of 190 prevalent cancer cases (5.9%) in the sample with 22.6% of them being breast cancer (See Figure 1). Colon and uterine cancer constituted 5.8% and 5.3% of the total prevalent cancer cases. The most common hormone used was estrogen with a negligible percentage on progestin. See Figure 2 for details of distribution.

The comparative analysis of prevalent cancer cases and controls suggest that the cases were on average 6 years older (Table 3). The two groups were different in the following dietary practices: total fat intake (p = 0.0267), total vitamin E intake (p = 0.0349), Lycopene (p < 0.0001), and % calories from alcohol (p = 0.029). Physical activity as measured by Home and Yard activities differed significantly between the two groups (p = 0.0433). There was a significant difference in the mean age of onset for use of birth control pills between the two groups (p = 0.0050). The prevalence of hypertension (p = 0.030) and Type 2 diabetes mellitus (p = 0.0196) was significantly higher in those with prevalent cancer than in those without cancer.

Similar to Table 2, Table 3 provides comparisons of breast cancer cases and controls. Similar to prevalent cancer, the prevalent breast cancer cases were 7 years older than the controls (p < 0.0012). There was no significant difference between the two groups on the dietary practice measures. The post menopause status was more frequent in the breast cancer cases than in controls (94.6% *vs.* 73.4%; p = 0.0037).

To examine the joint effect of HRT and menopause, the three subgroups (pre-menopausal women without HRT, post-menopausal women not on HRT and post-menopausal women on HRT) were compared with respect to the factors and outcome measures considered in this study (Table 4). Assessing differences across the three groups the following were significant: age (p < 0.0001), BMI (p = 0.0063), vegetable intake (p < 0.0001), % calories due to fat (p < 0.0001), total fat intake (p < 0.0001), total dietary fiber (p = 0.0006), Beta-Carotene (p = 0.0209), total vitamin E (p < 0.0001), vitamin C (p = 0.0053), Lycopene (p < 0.0001), % calories due to alcohol (p = 0.0008), age of onset of birth control pill (p < 0.0001), duration of birth control pill use (p < 0.0001) and all five measures of physical activity (total score (p < 0.0001), Home & Yard (p < 0.0001), active living(p < 0.0001), work index (p = 0.0103) and sport index (pp < 0.0001). The joint effect of menopause and HRT was significantly associated with education level (p < 0.0001), family income (p < 0.0001), ever taking birth control pills (p < 0.0001), type 2 diabetes mellitus (p < 0.0001), hypertension (p < 0.0001), prevalent CVD (p < 0.0001), prevalent breast cancer (p = 0.0223) and prevalent cancer (p = 0.0008).

Prevalent cancer was associated with age (OR: 1.04; 95% CI: 1.03, 1.06), and family history of cancer (OR: 1.55; 95% CI: 1.07, 2.23) in age-adjusted models. However, for breast cancer it was only associated with age (OR: 1.05; CI: 1.02, 1.08). None of the demographic and socioeconomic measures, and clinical and behavior risk factors were independently associated with the outcome measures (Cancer and breast cancer). Data is not presented in this paper. In unadjusted models the odds of prevalent cancer was significantly higher in post-menopausal women who were not on HRT (OR: 3.33; CI: 2.09, 5.32) and those who were on HRT (OR: 2.31; CI: 1.34, 4.00) compared to women who were pre-menopausal (Table 5). However, in the age-adjusted models, the odds of prevalent cancer was higher in post-menopausal women who were not on HRT (OR: 1.97; CI: 1.15, 3.38) but not in women who were on HRT (OR: 1.53; CI: 0.85, 2.75) when compared to pre-menopausal women. The earlier association between prevalent cancer and the joint effect of menopause and HRT was diminished when age and family history of cancer were adjusted for as covariates. However, in the case of prevalent breast cancer, post-menopausal women who were not on HRT showed significantly higher odds compared to pre-menopausal women in both the unadjusted (OR: 7.59; CI: 1.81, 31.82) and age-adjusted models (OR: 4.85; CI: 1.03, 22.85).

## 4. Conclusions

In this study, several factors in the following domains of measurement, Demographic and Socioeconomic, Clinical and Behavioral Risk Factors, were identified as factors that were associated with prevalent cancer and breast cancer. Also, several of these factors were significantly associated with the joint effect of menopause status and HRT. Though collection of cancer-related data was limited in the JHS cohort, the estimated prevalence of cancer in African American women represented by the JHS was 5.9%, slightly more than half of the prevalence of cardiovascular disease (9.5%) in the same sample population. This finding seems consistent with the ratio of CVD and cancer mortality in the State of Mississippi, the two most common causes of mortality in the state. Of the types of cancers in this sample, the most prevalent was breast cancer, which represented 22.6% of all cancers cases. These findings were consistent with the literature that refers to breast cancer as the most common form of cancer and the second leading cause of death among women in the United States [1].

The unadjusted comparison of dietary practices, total fat intake, total vitamin E intake, Lycopene and % calories from alcohol significantly differ between cancer and non cancer cases. However, these significant associations were attenuated in the age adjusted logistic models. This study does not support earlier findings in the literature that certain lifestyles, such as dietary practices, tobacco use, alcohol consumption, weight gain, and physical activity, as well as high blood pressure, considered traditional CVD risk factors, may be linked to the development of both CVD and cancer (and particularly breast cancer). However, given the small number of cancer cases, it is important to note that the study findings underscore that there is little known association between dietary practices and cancer in African American women [17–19]. Thus, this warrants further investigation. The mean % calories due to fat in the study sample was high for all subgroups (cancer *vs.* non-cancer), (breast cancer *vs.* non-breast cancer) and (pre-menopause, post-menopause without HRT and post-menopause with HRT) based on the cut-point of greater than 30% of total energy consumed set forth by the National Institute of Health’s National Heart, Lung, and Blood Institute [20].

Physical activity measured by home and yard activities was significantly associated with cancer in the unadjusted models. However these associations were attenuated in the age adjusted models. The mean age of the onset of use of birth control pills, and two classical CVD risk factors, hypertension and Type 2 diabetes mellitus, were also significantly related to cancer. However, these significant findings were not evident for prevalent breast cancer. A plausible explanation could be the low prevalence of breast cancer (1.1%) in the study sample.

Since menopause, but not HRT, was independently associated with cancer, and the joint effect of menopause and HRT suggested that post-menopausal women who were not on HRT had higher odds ratios of cancer (and breast cancer) compared to pre-menopausal and post-menopausal women who were on HRT, further investigation of these findings is warranted. The pursuit of the recommended further studies in this area of scientific investigation is very important for the following reasons. The number of cancer and breast cancer cases in this study was relatively small and the findings could be validated or challenged with studies of larger numbers of cancer or breast cancer cases. Secondly, [21] reported that the assessment of the risks and benefits of HRT relative to cancer, CVD and other chronic diseases in African Americans and other non-white women is very limited given their lack of or minimal participation in clinical studies on HRT.

### Perspective

Though data for classifying cancer and especially breast cancer was limited in the Jackson Heart Study compared to extensive data on CVD outcomes and risk factors, this study provides insight into the potential of ascertaining additional data specific to cancer etiology, progression and survival, given the extensive data on biological and psychosocial determinants of cancer morbidity and mortality as outcome measures. The reproductive history coupled with repeated data collection on medication use, provides a foundation for future studies of cancer that utilizes the Jackson Heart Study data.

Given that the Jackson Heart Study is a longitudinal study, it provides scientific resources for future recommended investigations that may examine the joint effects of menopause and HRT on the risk of developing cancer (breast cancer) in African American women.

## Figures and Tables

**Table 1.** Baseline characteristics of african american women (35–84 years) in JHS (n = 3,202).

**Characteristics**	**N (%)**
Age (years)	56 ± 11
BMI, (Kg/m^2^)	32.8 ± 7.4
Obesity (%)	60.3
***Education Level (%)***	
Less than HS	18.4
High School/GED	20.6
Greater than HS but less than BA/BS	27.7
Bachelor Degree or Higher	33.3
***Family Income (%)***	
Low	17.3
Lower- Middle	27.0
Upper Middle	29.9
Affluent	25.8
Family History of Cancer (%)	1,576 (59.8)
Total number of pregnancies	3.5 ± 2.5
Total number of live born children	3.2 ± 2.2
Menopausal Post (%)	2,338 (73.7)
HRT Use (%)	710 (22.6)

**Table 2.** Comparative analysis of socio-demographic, behavioral and clinical risk factors by prevalent cancer status.

**Characteristics**	**Non-Cancer (n = 3,012)**	**Cancer (n = 190)**	**P-Value**
Age (years)	56 ± 11	62 ± 11	<0.0001
BMI, (Kg/m^2^)	32.8 ± 7.4	31.9 ± 6.9	0.0914
Obesity	60.5	56.1	0.2331
***Education Level***			***0.4203***
Less than HS	18.40	20.53	
High School/GED	20.59	19.47	
Greater than HS but less than BA/BS	27.68	31.58	
Bachelor Degree or Higher	33.33	28.42	
***Family Income***			***0.7465***
Low	17.28	19.23	
Lower- Middle	26.98	29.49	
Upper Middle	29.92	26.92	
Affluent	25.82	24.36	
Current Smoking, Yes	10.33	8.47	0.4126
Heavy Alcohol Use (%)	5.71	6.52	0.8055
Fruit Intake (grams/day)	1.5 ± 1.1	1.5 ± 0.8	0.8412
Vegetable Intake (grams/day)	1.4 ± 0.6	1.3 ± 0.5	0.0635
% Calories due to Fat	34.9 ± 7.2	34.1 ± 7.0	0.1100
Amt. of Fat Intake (grams)	76.4 ± 47.5	69.6 ± 39.2	0.0267
Total Dietary Fiber (grams)	14.7 ± 7.0	14.1 ± 5.5	0.1489
Beta-Carotene (Mcg)	3,288 ± 1,545	3,190 ± 1,512	0.4043
Total Vitamin E (Mg)	66.1 ± 107.9	85.7 ± 121.9	0.0349
Vitamin C (Mg)	188.9 ± 190.2	210.7 ± 207.4	0.1344
Lycopene (Mcg)	4,209 ± 4,989	3,253 ± 2,921	< 0.0001
%Calories from Alcohol	0.5 ± 2.0	0.3 ± 1.2	0.0291
Post Menopausal (%)	72.7	89.0	< 0.0001
HRT Use, Yes (%)	22.8	21.3	0.6402
***Types of HRT***			***0.6402***
Estrogen	74.5	67.1	0.2987
Progestin	1.1	0.0	0.7143
Estrogen & Progestin	12.6	8.2	0.1767
Family History of Cancer	59.0	71.7	0.0019
Physical Activity Total	8.1 ± 2.6	7.3 ± 2.6	< 0.0001
Active Living	2.1 ± 0.8	2.0 ± 0.8	0.2479
Home and Garden	2.3 ± 0.6	2.2 ± 0.6	0.0443
Sport Index	2.1 ± 1.2	2.0 ± 1.2	0.1418
Work Index	2.6 ± 0.7	2.6 ± 0.6	0.7368
Type Two Diabetes	20.12	27.37	0.0196
Hypertension	66.14	73.91	0.0300
Prevalent CVD	9.25	12.77	0.2331
Age start taking birth control pills	22 ± 5	23 ± 5	0.0050
Age stop taking birth control pills	30 ± 7	30 ± 6	0.9026
Years you have used birth control	8 ± 6	7 ± 5	0.0710

**Table 3.** Comparative analysis of socio-demographic, behavioral and clinical risk factors by prevalent breast cancer.

**Characteristics**	**Non-Breast Cancer (n = 3,165)**	**Breast Cancer (n = 37)**	**P-Value**
Age, years	56 ± 12	63 ± 10	0.0012
BMI, (Kg/m^2)^	32.8 ± 7.4	32.4 ± 6.8	0.7548
Obesity	60.3	62.2	0.8207
***Education Level***			***0.9104***
Less than HS	18.65	16.22	
High School/GED	20.46	21.62	
Greater than HS but less than BA/BS	27.85	32.43	
Bachelor Degree or Higher	33.05	29.73	
***Family Income***			***0.8961***
Low	17.44	20.0	
Lower- Middle	27.30	23.33	
Upper Middle	29.70	26.67	
Affluent	25.57	30.0	
Current Smoking, Yes	10.25	2.70	
Heavy Alcohol Use (%)	5.76	0.0	0.0707
Fruit Intake (grams/day)	1.5 ± 1.1	1.6 ± 0.7	0.3092
Vegetable Intake (grams/day)	1.4 ± 0.6	1.3 ± 0.4	0.0776
% Calories due to Fat	34.9 ± 7.2	34.6 ± 7.8	0.8336
Amt. of Fat Intake (grams)	75.8 ± 47.0	80.8 ± 47.4	0.5305
Total Dietary Fiber (grams)	14.7 ± 6.9	14.8 ± 5.2	0.8968
Beta-Carotene (Mcg)	3286 ± 1544	3056 ± 1239	0.3799
Total Vitamin E (Mg)	67.0 ± 108.6	74.5 ± 1193.2	0.6813
Vitamin C (Mg)	189.3 ± 190.6	227.1 ± 224.9	0.2437
Lycopene (Mcg)	4144 ± 4900	3663 ± 2250	0.2250
%Calories from Alcohol	0.5 ± 1.9	0.3 ± 1.1	0.4515
Post Menopausal (%)	73.4	94.6	0.0037
HRT Use, Yes (%)	22.7	13.5	0.1853
***Types of HRT***			
Estrogen	74.2	61.5	0.1637
Progestin	1.0	0.0	0.3710
Estrogen & Progestin	12.3	0.0	0.2732
Family History of Cancer	59.7	65.5	0.5269
Physical Activity Total	8.1 ± 2.6	7.7 ± 2.0	0.4029
Active Living	2.1 ± 0.8	2.0 ± 0.8	0.6252
Home and Garden	2.3 ± 0.6	2.3 ± 0.6	0.8008
Sport Index	2.1 ± 1.2	2.0 ± 1.2	0.6959
Work Index	2.6 ± 0.7	2.5 ± 0.7	0.5325
Type Two Diabetes	20.54	27.78	0.2858
Hypertension	66.56	80.56	0.0764
Prevalent CVD	9.44	5.56	0.4167
Age start taking birth control pills	22 ± 5	24 ± 5	0.0953
Age stop taking birth control pills	30 ± 7	31 ± 5	0.3383
Years you have used birth control	8 ± 6	7 ± 5	0.3157

**Table 4.** Relation of risk factor correlates and the joint effect of menopause and hrt in jhs (3,142)*.

**Characteristics**	**Pre-Menopause (n = 835)**	**Post-Menopause w/out HRT (n = 1,673)**	**Post-Menopause w/HRT (n = 634)**	**P-Value**
Age, years	45 ± 8	61 ± 10	58 ± 9	<0.0001
BMI, (kg/m^2^)	33.3 ± 8.3	32.9 ± 7.2	32.0 ± 6.7	0.0063
Obesity, Yes	61.44	61.12	56.99	0.1463
***Education Level***				<0.0001
Less than High School	6.73	25.52	14.80	
High School/GED	15.63	23.67	19.21	
Greater than HS but less than BA/BS	36.90	23.55	28.35	
Bachelor Degree or Higher	40.75	27.26	37.64	
***Family Income***				<0.0001
Low	16.45	20.75	10.70	
Lower- Middle	21.70	31.20	23.89	
Upper Middle	35.60	25.86	31.37	
Affluent	26.24	22.19	34.05	
Current Smoking, Yes	10.71	10.17	8.85	0.4860
Heavy Alcohol Use	6.3	6.0	3.4	0.5325
Fruit Intake	1.4 ± 1.1	1.5 ± 1.1	1.5 ± 1.1	0.1369
Vegetable Intake	1.5 ± 0.7	1.4 ± 0.6	1.4 ± 0.6	<0.0001
% Calories due to Fat	36.4 ± 7.2	34.2 ± 7.0	34.4 ± 7.2	<0.0001
Amt. of Fat Intake	89.6 ± 53.1	70.5 ± 43.4	70.3 ± 38.3	<0.0001
Total Dietary Fiber	15.4 ± 7.1	14.4 ± 6.9	14.3 ± 6.1	0.0006
Beta-Carotene	3,183 ± 1,629	3,280 ± 1,477	3,408 ± 1,462	0.0209
Total Vitamin E	52.7 ± 95.9	66.9 ± 108.8	85.1 ± 120.4	<0.0001
Vitamin C	177.9 ± 175.0	187.5 ± 190.7	210.2 ± 208.1	0.0053
Lycopene	4,969 ± 5,998	3,827 ± 4105	3,754 ± 4,139	<0.0001
%Calories from Alcohol	0.7 ± 2.4	0.4 ± 1.8	0.4 ± 1.4	0.0008
Ever taken Birth Control, Yes	82.5	49.5	62.9	<0.0001
Age of Onset Use of Birth Control	20 ± 4	23 ± 5	23 ± 5	<0.0001
Duration of Use of Birth Control	9 ± 7	7 ± 6	8 ± 6	<0.0001
Family History of Cancer	51.72	63.50	60.34	<0.0001
Physical Activity Total	9.0 ± 2.3	7.6 ± 2.6	8.2 ± 2.5	<0.0001
Active Living	2.2 ± 0.8	2.0 ± 0.8	2.1 ± 0.8	<0.0001
Home and Garden	2.4 ± 0.6	2.2 ± 0.6	2.2 ± 0.5	<0.0001
Sport Index	2.2 ± 1.2	2.0 ± 1.2	2.2 ± 1.2	<0.0001
Work Index	2.6 ± 0.7	2.7 ± 0.6	2.6 ± 0.6	0.0103
Breast Cancer	0.84	2.09	0.94	0.0223
Cancer (General)	3.40	11.05	7.33	<0.0000
Type Two Diabetes	11.0	25.3	20.2	<0.0001
Hypertension	43.4	73.8	78.3	<0.0001
Prevalent CVD	4.1	12.7	8.1	<0.0001

* Women who were pre-menopausal and on HRT (n = 60) were excluded from the analysis.

**Table 5.** Association of prevalent cancer and breast cancer with the joint effect of menopause and hrt.

**Outcome**	**Models**	**Pre-Menopause**	**Post-Menopause w/out HRT**	**Post-Menopause w/ HRT**
Prevalent Cancer	I	1.00	3.33 (2.09,5.32)	2.31 (1.34,4.00)
	II	1.00	1.97 (1.15,3.38)	1.53 (0.85,2.75)
	III	1.00	1.76 (0.98,3.17)	1.54 (0.81,2.91)
Prevalent Breast Cancer				
	I	1.00	7.59 (1.81,31.82)	3.29 (0.64,17.03)
	II	1.00	4.85 (1.03,22.85)	2.32 (0.42,12.74)
	III	1.00	3.56 (0.73,17.43)	1.79 (0.30,10.60)

Model I: unadjusted; Model II: Age-adjusted; Model III: adjusted for Age and Family History of Cancer.
